# Nanostructured Biomaterials in 3D Tumor Tissue Engineering Scaffolds: Regenerative Medicine and Immunotherapies

**DOI:** 10.3390/ijms25105414

**Published:** 2024-05-16

**Authors:** Athina Angelopoulou

**Affiliations:** Department of Pharmacy, School of Health Sciences, University of Patras, 26504 Patras, Greece; angelopoulou@upatras.gr or angelopoulouathina@gmail.com

**Keywords:** tissue engineering, cancer, immunotherapy, 3D scaffolds, biomaterials

## Abstract

The evaluation of nanostructured biomaterials and medicines is associated with 2D cultures that provide insight into biological mechanisms at the molecular level, while critical aspects of the tumor microenvironment (TME) are provided by the study of animal xenograft models. More realistic models that can histologically reproduce human tumors are provided by tissue engineering methods of co-culturing cells of varied phenotypes to provide 3D tumor spheroids that recapitulate the dynamic TME in 3D matrices. The novel approaches of creating 3D tumor models are combined with tumor tissue engineering (TTE) scaffolds including hydrogels, bioprinted materials, decellularized tissues, fibrous and nanostructured matrices. This review focuses on the use of nanostructured materials in cancer therapy and regeneration, and the development of realistic models for studying TME molecular and immune characteristics. Tissue regeneration is an important aspect of TTE scaffolds used for restoring the normal function of the tissues, while providing cancer treatment. Thus, this article reports recent advancements in the development of 3D TTE models for antitumor drug screening, studying tumor metastasis, and tissue regeneration. Also, this review identifies the significant opportunities of using 3D TTE scaffolds in the evaluation of the immunological mechanisms and processes involved in the application of immunotherapies.

## 1. Introduction

Cancer therapeutics consisting of nanostructured materials are commonly based on research about the molecular, biological and immunological effects on 2D in vitro cell or tissue cultures [1,2,3]. Cancer biology and immunology of the primary cells and cell lines involved have expanded the understanding and development of new therapeutic strategies for diagnosis, prevention and treatment [4,5]. Studies on the molecular signaling pathways have elevated our comprehension of drug effects in combination with varied nano-biomaterials and external stimuli [6,7,8]. The vast majority of the drug combinations researched have exhibited significant antitumor effects in 2D in vitro experiments and in vivo animal models. Despite this, the therapeutic outcomes of clinical trials are insufficient for promoting the therapeutic effects of these drug combinations and undesirable toxicity levels have developed, resulting in a limited number of nanostructured drug formulations reaching clinical practice [9]. A possible explanation could be the weak relevance of the in vitro and in vivo models to human tumor tissues due to the dynamic biological, chemical and mechanical characteristics of the tumor microenvironment (TME) in human tissues [10,11]. Mechanical aspects of the TME, such as cell–cell and cell–ECM (extracellular matrix) tension, increasing ECM stiffness and solid stress (due to the expanding tumor mass), hydrostatic pressure, fluid shear stress and interstitial fluid pressure are the driving forces behind high levels of TME heterogeneity. The effect of TME heterogeneity has proved to be essential for tumor cells epithelial-to-mesenchymal transition (EMT), stem-like phenotype, abnormal metabolic alterations and invasiveness [12,13,14]. Significant effort has been devoted to the in vivo understanding of TME heterogeneity in order to overpower the limitations of the TME and increase drug accumulation and tumor site specificity. In this context, the development of in vivo targeting mechanisms to achieve deep tumor penetration in animal models has evolved dramatically. The intense research interest, both in vitro and in vivo, devoted to the development of responsive nanostructured medicines for use in the TME has resulted in a better understanding of the fundamental pathways related to the immune reprogramming of the TME [14]. TME heterogeneity is an intense research field and 2D in vitro and in vivo animal models have definitely provided a significant amount assistance to elucidating the related mechanisms. However, TME heterogeneity in cancer patients leads tumor cells to undergo a phenotype selection associated with genetic rearrangement, M2 anti-inflammatory polarization and genomic instability which affect the ECM, blood vessels and tumor stroma development [12,13,14].

The evaluation of nanomedicines has been associated with 2D cell cultures that provide an insight into biological mechanisms at the molecular level through primary cells and cell lines of a specific phenotype [15,16]. However, the main drawback of such cultures is the reduced number of cell–cell interactions, the lack of ECM, the limited or no access to metabolic gradients and the absence of the mechanically dynamic aspects of the TME [10]. Additionally, critical aspects of the TME’s interaction with the whole organism are provided by the study of animal xenograft models that have proved to be powerful tools in simulating human tumors. The main drawback of animal models is the inadequate reproduction of the human tumor environment and its interactions. These hallmarks are being addressed by the development of patient-derived tumor models that are studied in immunosuppressive SCID mice in an attempt to reflect the human tumor’s function [11].

**Figure 1:**
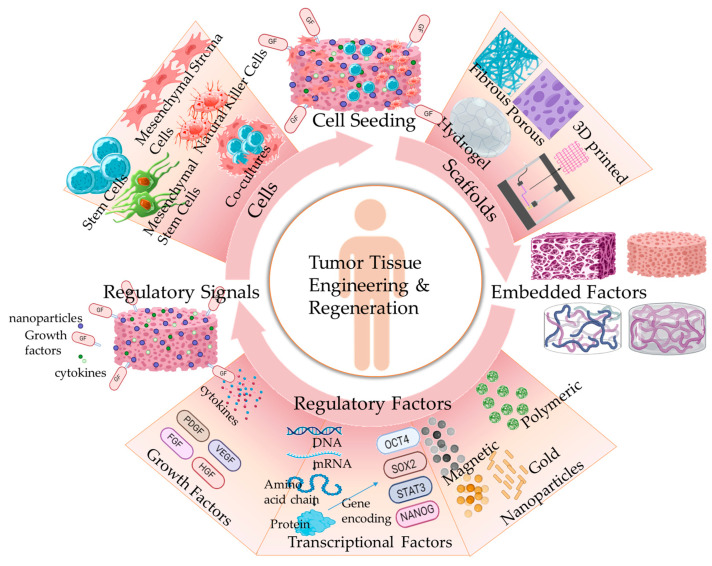
Representation of basic characteristics of tumor tissue engineered (TTE) scaffolds in combination with varied nanostructured materials, regulatory and growth factors and cell types. (Created with the assistance of BioRender.com https://app.biorender.com/user/signin?illustrationId=6156d45891063d00af8af51d (accessed on 27 April 2024 up to 28 April 2024) and Microsoft ppt (software version number: Microsoft Office Professional Plus 2016)).

In order to provide more realistic models that can histologically reproduce human tumors, novel methodologies of co-culturing cells with varied phenotypes have been applied, in combination with the development of 3D tumor spheroids or tumoroids [5,11]. The co-culture and development of multicellular tumoroids can take place in suspensions or in structural matrices of varied compositions. In this respect, Girard et al. [17] developed a 3D nanofibrous scaffold based on biocompatible polymers that could be used as a platform for the culture of tumor cells and the growth of tumoroids. Within the 3D scaffold, tumor cells were engaged in EMT and the 3D tumoroids expressed higher drug resistance in comparison to monolayer cell cultures. Tumoroids, either grown in suspensions or in matrices, are 3D structures that mimic the biochemical and mechanical characteristics of the tumor tissue environment [18]. In novel approaches in the form of 3D tumor models, tumor tissue engineering (TTE) scaffolds have been developed based on complex matrixes, including hydrogels, 3D bioprinted materials, biomaterials, synthetic polymers, decellularized matrices, microspheres, fibrous and nanostructured particles. The TTE scaffolds (Figure 1) are designed to mimic the ECM of the TME, and thus are characterized by solid porous or fibrous structures for the incorporation and adhesion of tumor cells and growth factors [19]. Thus, they present an effective adhesion platform for the growth of tumoroids, the evaluation of mechanical and molecular TME characteristics and the development of diagnostic methods [20].

In the past decade, TTE scaffolds have found applications in the development of 3D tumor models, including spheroids, organoids and organ-on-a-chip (OoC) models that have been researched for the study of cancer development, biology and immunology [21]. The 3D scaffolds are composed of (i) natural polymers, such as collagen and chitosan, (ii) acellular matrices produced through the decellularization of allogeneic or xenogeneic tissues to provide an intact ECM with no cellular components, (iii) synthetic biodegradable and biocompatible polymers or (iv) mixed combinations of the above [22]. The TTE scaffolds are designed to present biomimetic platforms of a human tumor ECM. The key characteristics that TTE scaffolds must have are a porous microstructure, degradability, mechanical integrity and strong bioadhesion for the attachment of tumor cells, stromal cells and stem cells [23]. The TTE scaffolds have also been utilized in wound healing for lesions after surgical resection, since they support cellular adhesion, migration and differentiation thus promoting cell–cell interactions with the surrounding tissues. The OoC models are TTE biomodel platforms that combine microfluidic technologies and cell cultures in order to mimic the physicochemical properties of the TME, in contrast to organoids that are mainly sustained in static conditions. There is a huge variety of OoC biomodels, including lung, gut, liver, brain, breast and multi-organ chips [24].

Moreover, the most acknowledged and researched applications of TTE scaffolds are within targeted drug delivery and immunotherapy [25,26]. The high heterogeneity and abnormal vasculature of the TME promote disturbed blood flow accompanied with distorted hydrostatic and osmotic pressure in the blood vessels, further promoting neovascularization and tumor hypoxia. The dynamic nature of the TME has a profound effect on impaired cellular signaling promoting the generation of multiple gene mutations, the M2 anti-inflammatory phenotype of macrophages and immunosuppression [14]. The nature of the TME imposed important obstacles in the effective delivery of therapeutics at the tumor site, promoting inadequate tumor treatment. Among novel therapeutic strategies, the TTE scaffolds have been studied for drug delivery, gene therapy and cellular engineering. A characteristic example of scaffolds are hydrogels that are injected at the site of tumor resection for sustained drug release and the inhibition of tumor metastasis [27]. Other applications of hydrogel scaffolds include injection at the tumor site for sustained release and effective tumor suppression [22].

An attractive research field is the combination of nanostructured materials with TTE scaffolds to enhance the delivery of multiple bioactive agents (such as drugs, growth factors, genes, plasmids, antigenes). Moreover, nanomaterials are embedded in TTE scaffolds in order to provide them with tunable mechanical properties, and as contrast agents for real-time monitoring of the engineered tissues [28]. Furthermore, microporous scaffolds of biodegradable polymers, such as PLGA, have been evaluated in animal models for sustained drug release and bone tissue regeneration [29,30]. Tissue regeneration is an important aspect of using TTE scaffolds to restore the normal function of the tissues while providing cancer treatment. TTE scaffolds are designed as a reservoir to entrap cells, such as stem cells, chimeric antigen receptor (CAR)-T cells and natural killer (NK) cells, so that effective cell therapies can be carried out at the tumor site. In such applications TTE scaffolds are supplementary as they promote cell–cell interactions between tumor cells and cells from the host’s immune system. CAR-T cells are responsible for the identification of tumor-associated antigens; thus, in TTE scaffolds, upon interaction with tumor cells CAR-T cells would occupy a co-stimulatory domain to activate host immune T cells [31]. NK cells are utilized in TTE scaffolds in combination with cytokines and transcription factors to indirectly strengthen the host’s immune system by activating innate immune functions. Moreover, stem cells are often applied in TTE scaffolds immunotherapy since they can differentiate into NK cells and provide a significant amount of feedback. The important role of stem cells in tissue engineering is based on their self-renewal ability, and their capacity to proliferate and differentiate into multiple cell types modulating the host’s immune system [32].

Other important applications that have emerged for TTE scaffolds are in the development of tissue-engineered metastasis models in order to study the cancer biology of tumor metastasis. The biological and physical mechanisms underlying migration and invasion of metastatic tumor cells and of circulating tumor cells are poorly understood. Physical and mechanical factors such as the hemodynamic forces of blood flow, interstitial fluid flow, hydrostatic pressure and fluid shear stress strongly influence the fate of metastatic and circulating tumor cells. Thus, the development of a deeper understanding of tumor metastasis is based on TTE models that can effectively mimic the TME and the cell–ECM, cell–host cell and cell–tumor cell interactions [33]. Great support in this direction is provided by tissue-engineered bioreactors that simulate the biochemical and biomechanical processes in controlled conditions for tissue development. Bioreactors offer a realistic model for cell expansion, recellularization of the TTE scaffolds and nutrition and metabolism of the developed tissues for effective cellular adhesion. Thus, optimized parameters to model the dynamic continuous flow conditions are used for the development of 3D tissues. Novel bioreactor models have been developed expressing the complexity of tumor metastasis under vascular physical conditions. These bioreactors signify the importance of hemodynamic physical forces in the study of tumor metastasis [34,35]. TTE scaffolds are not applied as a single therapy in cancer treatment but as an adjuvant for efficient drug delivery, gene and cell therapy and immunotherapy. The purpose of this review is to provide insight into the role of tissue engineering in regenerative medicine, a scientific field known as TERM, to improve therapeutic strategies for cancer treatment. The review will outline the capabilities of tissue engineering in tumor medicine by providing novel cancer treatment alternatives and evaluating its effectiveness in immunotherapies.

## 2. Tissue Engineering in Cancer Therapeutics: Regenerative Medicine

The potential applications of regenerative medicine in cancer therapy include the scientific fields of cell and gene therapy and tissue engineering [31,36]. Regenerative medicine aims to restore or replace the damaged tissues or organs, and tissue engineering plays a crucial role in achieving this goal. Tissue engineering utilizes the principles of engineering and biology to design scaffolds, which act as temporary platforms to support the growth and development of cells, and guide the regeneration of new healthy tissues, while providing restoration or regeneration to the damaged tissues. By combining the principles of regenerative medicine and tissue engineering, researchers are able to develop innovative approaches with the ultimate goal of combining cells, biomaterials and growth factors to provide effective therapeutic outcome and restore tissues normal functions [24,25,37].

In cancer therapy, tissue engineering and regenerative medicine (TERM) represent a highly multidisciplinary research field. TERM combines biomimetic scaffolds and nanostructured materials with the therapeutic strategies of drug delivery, cancer ECM modeling and immunotherapy including stem cells therapy and gene therapy [25]. The TTE scaffolds have found successful applications in controlled and targeted antitumor therapy. First, TTE scaffolds can incorporate nanostructures, drugs and bioactive agents for effective delivery to and sustained release at the tumor site with limited toxicity in healthy tissues, in comparison to conventional chemotherapeutic strategies. Secondly, TTE scaffolds provide an effective platform for stem cells’ attachment, including mesenchymal stem cells and adipose-derived stem cells, that are able to differentiate and proliferate to varied cell lineages, depending on the growth factors co-delivered for restoring the structure and function of the lost tissue [28]. Despite the many advantages, TERM faces important limitations concerning the poor mechanical properties of the TTE scaffolds and weak cellular adhesion resulting in ineffective differentiation. In this aspect, the presence of nanostructured materials has been employed to enhance the mechanical properties of TTE scaffolds. For example, metallic nanoparticles like gold or silver can improve the mechanical strength of scaffolds, making them more suitable for load-bearing applications [28,38].

Moreover, ceramic nanoparticles like hydroxyapatite can improve the stiffness and strength of scaffolds for bone tissue engineering [39]. The utilization of ceramic nanoparticles, such as bioactive glass ceramic nanoparticles (n-BGC) and bioresorbable nanoceramics, has been applied in therapeutic strategies for bone tissue engineering and dentin regeneration. These nanoparticles can enhance cellular activities, promote bone regeneration and improve the mechanical properties of scaffolds. Bioactive glass nanoparticles (nBGs) have been studied for their incorporation in TTE scaffolds for bone tissue applications due to their antibacterial and angiogenic properties. The nBGs, owing to their biodegradability and metabolism, have a profound effect on the proliferation rate of osteoblasts, resulting in new bone formation. In a recent review by Aldhaher et al. [40], the tissue engineering applications of 3D hydrogel scaffolds with incorporated nBGs have been highlighted. Shoaib et al. [41] presented the synthesis of magnesium-doped nBGs (denoted as Mg-nBGs) for dual function in bone regeneration and antitumor drug delivery. The Mg-nBGs successfully promoted the formation of hydroxycarbonate apatite and inhibited the cell viability of osteosarcoma MG-63 cancer cells. Amine-functionalized copper (Cu)-doped nBGs have been used by Zhu et al. [42] in the 3D bioprinting of alginate dialdehyde and gelatin hydrogels in order to provide cell-ladened bioprinted scaffolds with enhanced cell compatibility for osteogenic differentiation. The 3D bioprinted scaffolds were evaluated against the human osteosarcoma MG-63 cell line in terms of cell adhesion and proliferation, and against the murine bone marrow-derived stromal ST2 cell line for its ability to stimulate angiogenic potential.

Gold nanoparticles (AuNPs) represent an important participant in the TERM scientific research field. In addition to providing mechanical support to the 3D scaffolds, Au nanoparticles represent ideal candidates for the delivery of drugs, growth factors and DNA or RNA genes to the cells. This way, the TTE scaffolds mimic the function of the ECM and enhance cellular and biological responses [43]. In a recent study, Radwan-Praglowska et al. [44] reported the development of 3D biomaterial scaffolds of poly(lactic acid), PLA, nanofibers. The PLA nanofibers were functionalized with nanohydroxyapatite and cross-linked in a chitosan–aerogel matrix. Nanoparticles, such as Au, Pt and TiO2, were embedded into the PLA nanofibers to enhance the mechanical properties of the scaffolds, stimulate cell proliferation and provide antibacterial effects. These scaffolds were evaluated in the development of bone tissue engineering. In another study by Zhang et al. [45], Au nanorods and nanostars were incorporated into porous gelatin 3D scaffolds to enhance photothermal cancer therapy. The 3D scaffolds showed a good biocompatibility, improved photothermal efficiency and increased cytotoxicity against human cervical carcinoma (HeLa) cancer cells.

Moreover, AuNPs have been used in gene delivery scaffolds for DNA plasmids by Tencomnao et al. [46]. In this study, gold/cationic polymer DNA scaffolds were developed and their transfection efficiency was evaluated using a human lung adenocarcinoma epithelial cell line (A549) and a human cervical cancer cell line (HeLa). The gold/polymer nanoscaffolds provided enhanced transfection efficacy in relation to the polymeric alternative scaffolds. Apart from gene delivery and photothermal therapy, AuNPs offer great potential as a participant in 3D TTE scaffolds for TERM since they can be applied in nuclear targeting and prevent cell division in human oral squamous cell carcinoma cells [47]. In a recent study, pegylated AuNPs incorporated in mesenchymal stem cells (MSCs) affected the MSCs’ migration rate and resulted in effective colonization and regeneration in fibrin and poly(caprolactone)-based scaffolds [48].

Stem cells, adipose-derived stem cells and MSCs represent the crucial backbones of TERM research and applications due to their elevated proliferation rate and capacity to differentiate into multiple tissue-specific cells [49]. Furthermore, stem cells are able to secrete varied trophic factors that can stimulate and promote immune responses and the cellular microenvironment for effective tissue regeneration [50]. In TTE, stem cells are applied in combination with 3D scaffolds and growth factors, such as fibroblast (FGF), epidermal (EGF) and platelet-derived (PDGF) growth factors, in order to create an effective microenvironment for differentiation. The main applications of stem cells in TTE are in combination with drug delivery systems for effective dual antitumor therapy and tissue regeneration. In this concept, 3D-printed hydrogels were studied by Liu et al. [51] for the targeted delivery of doxorubicin at the tumor site and the stimulation of tissue regeneration. In this study, 3D-printed alginate–gelatin hydrogel scaffolds were coated with polycaprolactone (PCL) and polydopamine (PDA). The release of doxorubicin and subsequent photothermal therapy inhibited tumor growth in breast cancer cells and the hydrogel scaffold promoted wound healing by enhancing the viability and proliferation of adipose-derived stem cells. An important potential use of stem cells in TTE is their application in multi-functional implanted scaffolds for preventing postoperative recurrence and distant metastases of tumors providing a key combination of tumor therapy and wound healing after surgery [52]. In this context, 3D-printed TTE scaffolds were developed by Zhang et al. [53] using gelatin bioinks for effective tumor treatment and tissue regeneration. The Pt(IV) prodrugs that were incorporated into the 3D scaffolds provided a significant inhibition of tumor growth against 4T1 breast cancer models. Simultaneously, the local recurrence and distant metastases were sustained and the ordered porous structures promoted cell attachment and the proliferation of normal cells. Thus, the transportation of nutrients was supported and new tissue growth was promoted to repair tissue defects in tumor resection sites.

### 2.1. Breast Cancer

Tissue engineering and regenerative medicine hold promising applications in the study of breast cancer biology and drug responses through the development of 3D tissue models. These 3D models provide a platform that mimics the breast TME to study cell–cell and cell–material interactions, in order to develop effective treatment strategies for the study of the fundamental molecular mechanisms for tumor progression, invasion and metastasis [54]. By engineering 3D tissue models, researchers can better understand the behavior of breast cancer cells and their responses to drugs, since it is possible to recapitulate the dynamic 3D ECM and the basic cellular interactions for tumor progression (Table 1) [55,56].

Three-dimensional TTE models have been used in drug screening in order to explore the differential effects of antitumor drugs and biological agents and their fundamental molecular mechanisms. In this context, Imamura et al. [57] studied the efficacy of common chemotherapeutic drugs, such as paclitaxel (PTX), doxorubicin (DOX) and 5-fluorouracil (5-FU), in 2D and 3D breast cancer models of six different cell lines and patient-derived primary cells grown as a patient-derived xenograft (PDX). The breast cancer cells developed multicellular spheroids (MCS) of dense or loose structure. The dense MCS expressed increased PTX and DOX drug resistance, hypoxia and elevated cleaved-PARP (poly(ADP-ribose) polymerase) expression levels suggesting that the dense spheroid structure protected the tumor cells from drug-induced apoptosis. In the case of dense MCS of BT-549 triple-negative breast cancer cells, the 3D models expressed lower Ki-67 expression levels than the 2D cultures, signifying that the G0-dormant subpopulation is responsible for the increased drug resistance.

Moreover, in the case of BT-474 (ER-negative/HER2-amplified breast cancer cells) dense 3D models, an anti-apoptotic TME was developed, since decreased expression levels of caspase-3 were detected. In the case of loose MCS, the expressed drug sensitivity of the 3D structures was at a similar level to the one expressed by the 2D cellular cultures. The study of PDX developed from patient-derived primary cells that expressed characteristics similar to in vivo-developed tumor models, while the dense 3D MCS better simulate the dynamic TME. Currently, PDXs represent effective preclinical strategies for evaluating standard-of-care therapies in 3D models that express common genetic stability with the human TME. The ex vivo culture PDX models (PDXEx) represent low-cost genetically relevant alternatives that generate most of the features of the original tumor. Eckhardt et al. [58] developed PDXEx tumor models originating from the cellular environment released from a mouse PDX tumor. The PDXEx and PDX models expressed close similarities in their tissue architecture, gene expression, cell signaling pathways and cellular differentiation. Thus, the 3D models retained a certain level of common inter- and intra-cellular interactions and physiological responses with the in vivo tumor models. For the development of the PDXEx, 3D magnetic bioprinting was used in inflammatory breast cancer, and IBC cells were used for the creation of iron nanoparticle-coated cells. Compact tumor spheroids were developed under the effect of a magnetic field which resulted in a levitating cellular mass. In both the PDX and PDXEx models, the developed spheroids were Ki67 positive indicating an active proliferation rate. The application of the PDXEx model in drug screening for IBC was assessed for anticancer agents used in standard clinical practice, such as eribulin, TAK228, doxorubicin, carboplatin, talazoparib, paclitaxel and gemcitabine. It was demonstrated that both the PDX and PDXEx models expressed similar drug response profiles.

The importance of 3D models in drug screening was also highlighted by Breslin et al. [59] in 3D models of HER2-positive breast cancer cell lines. The effects of neratinib (a tyrosine kinase inhibitor against HER2) and docetaxel were evaluated. The 3D models expressed an increased innate drug resistance that was enabled by the expression of proteins (Akt, Erk) and protein receptors (EGFR, pEGFR, HER3) involved in cell survival and drug transportation (p-glycoprotein, breast cancer resistance protein, BCRP). Moreover, the activity of drug-metabolizing enzymes, such as CYP3A4, was elevated in the 3D models in relation to the 2D cultures. The structure and spatial arrangement of the 3D TTE models were favorable for the development of cell–cell intra- and inter-cellular interactions highly affecting the expressed biological information. In another study by Gangadhara et al. [60], the drug response to endocrine agents (tamoxifen, fulvestrant) and trastuzumab was assessed for ER-positive/HER2-positive breast cancer 2D and 3D models. The drug sensitivity of the 3D models was decreased in relation to the 2D models. This effect was highly associated with an ECM-induced pathway switch from AKT to MAPK signaling, since in the 3D models a significant suppression of PI3K/AKT pathway activity was observed, while the MAPK signaling activity was elevated. The response of the 3D models to a single treatment with endocrine agents or trastuzumab resulted in further increase in MAPK pathway activity and reduced drug sensitivity. After inhibition of the MAPK pathway the drug sensitivity was restored signifying the role of MAPK/MEK signaling.

The underlying mechanisms associated with drug resistance in 3D TTE models were assessed by Uematsu et al. [61], who studied the drug sensitivity of 3D MCF7 breast cancer models to daunorubicin, docetaxel and arsenic disulfide. The 3D spheroids expressed increased drug resistance that was partially associated with P-glycoprotein function. An interesting study was presented by Muguruma et al. [62], which investigated the drug sensitivity of epirubicin (EPI), cisplatin (CDDP) and docetaxel (DTX) in 2D and 3D models of thirteen triple-negative breast cancer cell lines. All the 3D tumor models expressed significant drug resistance as evidenced by a cell viability assay. Drug sensitivity has also been examined by Liu et al. [63] in 3D collagen scaffolds which served as the matrix for cancer cell growth in a 3D environment. The MCF7 breast cancer cells and glioblastoma U118-MG cells that were attached to and proliferated in the 3D scaffold formed tumor spheroids and expressed elevated levels of reactive oxygen species (ROS) in relation to the 2D cultures. The reduced drug sensitivity of the developed compact spheroids against cisplatin was attributed to reduced drug uptake and the tumor-like microenvironment as evidenced by the elevated ROS levels.

Apart from drug screening, 3D TTE models have been used for the study of the role of biological interactions in tumor progression. Carter et al. [64] studied the effect of myoepithelial and luminal cells in the development of ductal carcinoma in situ (DCIS). A novel 3D model of the human breast duct bilayer was developed in collagen-based gels that promoted the reformation of the bilayer. HER2-expressing luminal cells were combined with myoepithelial cells in collagen gels for the development of ductal structures that formed intact bilayer structures with myoepithelial cells forming an outer ring around a hollow luminal center that expressed similar levels of biomarkers (P-cadherin, vimentin, CK8, EpCAM) to the human sections. The 3D models of the overexpression of HER2 in luminal cells were developed for the study of breast tumor progression through the destabilization of the bilayer and luminal filling of the center. The co-delivery of doxycycline and trastuzumab (HER2 targeted antibody) resulted in the suppression of the luminal filling. In another study by Fisher et al. [65], a 3D TTE model was developed to study the effect of growth factor gradients in breast cancer invasion. A 3D hyaluronic acid hydrogel scaffold crosslinked with matrix metalloproteinase (MMP)-cleavable peptides and conjugated with multiphoton labile nitrodibenzofuran (NDBF) was created in order to immobilize epidermal growth factor (EGF) gradients through a photochemical reaction. This model was used to evaluate the effect of varied EGFR expression levels on the invasive capacity of MDA-MB-231, MDA-MB-468 and MCF-7 breast cancer cell lines. Differential cellular responses were observed for the three breast cancer cell lines concerning cell invasion capacity in response to photopatterned EGF gradients. The moderate EGFR-expressing MDA-MB-231 cells showed increased invasion capacity, the MDA-MB-468-overexpressing EGFR exhibited reduced invasion and the MCF7 with low EGFR expression levels showed no invasion effect. Seemingly, the crosslinking density and pore size of the hydrogel were crucial aspects affecting hydrogel stiffness and further influencing the invasion capacity of the cells. In densely packed hydrogels more crosslinks need to be degraded for enough space to be created for the cellular transportation and invasion. The sensitivity of the models of the EGF inhibitor cetuximab was assessed, resulting in contradictory results in relation to cellular invasion that further highlighted the necessity of drug screening in combination with cell–cell and cell–material interactions.

The role of macropores in MDA-MB-231 breast cancer cells’ behavior was examined by Xiong et al. [66] in 3D scaffolds synthesized from bacterial cellulose (BC). The pore size of the 3D BC scaffolds provided an effective matrix for cell adhesion and proliferation, with good viability and penetration rate. Another important aspect of a 3D TTE scaffold structure is the combination of nano and submicro fibers that effectively mimic the tumor ECM. Recently, the effect of structural characteristics of 3D TTE scaffolds were examined by Yue et al. [67] in hydrogels with varied degree of crosslinked methacrylated gelatin (GelMA) to form microwell array systems of tunable stiffness. The stiffness range used in the study was 250 Pa–3 kPa, which was relevant to the ECM stiffness of physiological and pathological tissues. These microwell hydrogels were loaded with stromal cells in order to study the effect of ECM stiffness on tumor–stromal cell interactions and stromal cell adipogenesis. For this study, human triple-negative breast cancer cell lines, HCC1806 cells and MDA-MB-231 cells were attached to the microwells that contained the pre-adipocyte stromal cells, resulting in the formation of size-controlled tumor spheroids. The interaction of tumor spheroids with the stromal cells resulted in stiffness-dependent cellular differentiation and maturation. In high-stiffness tissue constructs, the adipogenesis was inhibited, in contrast to low-stiffness structures in which no significant inhibitory effect was observed. The development of mechanically tunable adipocyte-laden 3D scaffolds represented an interesting biomimetic platform for drug screening in a physiologically relevant environment.

The application of TERM in breast cancer enables the most accurate biological mimicry of the TME and cell–cell and cell–ECM interactions. TERM provides a promising means by which to understand the fundamental molecular mechanisms supporting tumor growth, progression and metastasis. The developed 3D TTE scaffolds are beneficial for the engineering of 3D tumor models that closely mimic the human TME, enabling scientists and researchers to study drug screening, sensitivity, metastasis and tumor biology. Though these biomimetic models align with biomolecules that can be used to investigate breast cancer biological processes, there is a need to design 3D TTE scaffolds that are more relevant to the TME’s micro milieu and breast cancer pathophysiology.

### 2.2. Bone Cancer

The development and application of 3D biomaterial scaffolds for bone tissue regeneration has been a scientific field with major research interest [68,69]. The high demand for bone repair materials is based on the various types of bone defects that have raised challenges for porous 3D scaffolds with established biocompatibility and biodegradability, tunable rigidity, strength and elasticity in supporting elevated osteoinduction and osteogenesis [70]. Furthermore, the 3D scaffolds should have certain characteristics, such as a lack of immunogenicity and toxicity, the inclusion of a biomimetic matrix for stimulated cell binding affinity and cellular responses and an ability to carry out localized and controlled delivery of drugs and bioactive agents. Nanostructured materials combined with 3D TTE scaffolds and genetic engineering have opened up new possibilities for enhanced cellular attachment in a biomimetic milieu in which cells are co-cultured with osteoblasts, stem cells and growth factors for improved osteogenic and biological activity (Table 2) [71].

The approaches of regenerative medicine in in vivo bone formation have presented major challenges in exploiting engineered cells and biomaterial scaffolds in order to support and promote the inherent regenerative capabilities of bone tissues, including osteogenesis and angiogenesis [72]. The advantages of different materials have been combined, such as bioceramics, metal oxides, polymer materials and tissue-engineered bone structures, in order to develop multifunctional scaffolds that effectively support living cells, and potentially stimulate bone repair and tissue regeneration [73,74]. The route of bone tissue regeneration is the engineering of cells, the introduction of these cells into the biomaterial scaffolds, the addition of nanostructured materials and growth factors for tunable properties and cell differentiation and the introduction of the final engineered scaffold into the host for effective bone regeneration [75]. Various cell types have been studied for these research applications, mainly including embryonic stem cells, pluripotent stem cells, bone marrow mesenchymal stem cells, adipose derived stem cells, osteoblasts and that have been genetically modified cells to express osteogenic factors [76].

The application of 3D biomaterial scaffolds in bone TTE is one of the most stipulating and highly developing research fields, since researchers need to combine tumor therapy and bone tissue regeneration. Yan et al. [77] studied the application of injectable materials as bone fillers for bone tissue repair and inactivation of the residual tumor cells in tumor-induced bone defects. For this study, α-tricalcium phosphate (α-TCP)/calcium sulfate (CS) biphasic bone cements were synthesized with incorporated Fe3O4/graphene oxide (GO) nanocomposites. The injectable magnetic bone cements promoted bone regeneration in rat bone-marrow-derived mesenchymal stem cells (rBMSCs) and in vivo in cranial defect models of rats, as evidenced by the relative bone gene expression levels. Moreover, an evaluation in osteosarcoma and lung metastasis tumor-bearing mice revealed effective tumor inhibition that was promoted by the hyperthermia effect of the magnetic bone cements. In a study by Pan et al. [78], gelatin nanofibrous (GF) 3D TTE scaffolds were developed with sintered mesoporous imidazolate framework 8 (ZIF8) nanoparticles and porphyrin-like macrocycles. The ZIF8 nanoparticles were loaded with Phenamil (Phe), an activator of bone morphogenetic protein pathways. The 3D scaffolds were studied for their application in the regeneration of bone tumor defects and the inactivation of residual tumor cells. The GF 3D TTE scaffolds promote bone morphogenetic protein 2 (BMP-2)-induced osteogenic differentiation under NIR (Near-Infrared Radiation) treatment and Phe release, as evidenced by evaluating the alkaline phosphatase (ALP) activities and the expression of bone-related genes (Col, RUNX2, BSP) on C2C12 myoblast cells. Moreover, the photothermal effect of the scaffold promoted the cell death of osteosarcoma MG-63 tumor cells in vitro and inhibited tumor growth in in vivo subcutaneous tumor models.

The development of 3D scaffolds for bone TTE with tunable bifunctional properties for bone regeneration and drug delivery has been significantly researched. The entrapment of nanostructured materials, such as graphene derivatives, metal oxides and nano-biomaterials, has been researched in order to develop 3D scaffolds with tunable properties [79,80]. Saber-Samandari et al. [81], developed bifunctional scaffolds composed of gelatin and akermanite that entrapped multiwalled carbon nanotubes and magnetic nanoparticles into a porous matrix. The engineered scaffolds presented an adequate biodegradation rate for the formation of an ECM during tissue regeneration, an increased protein adsorption rate and a good biocompatibility with osteoblast G292 cells. In another report by Jasemi et al. [82], porous calcium–zirconia scaffolds were prepared incorporating magnetic nanoparticles that were further coated with chitosan for bone tissue applications. The 3D scaffolds were biocompatible and had satisfactory mechanical and physical properties, and no toxic effects were reported upon treatment with bone marrow stem cells. Moreover, the scaffolds expressed an increased proliferation rate and bone regeneration ability.

The application of nanoparticle-enriched 3D printed biomaterial scaffolds was assessed by Dong et al. [83] for osteosarcoma treatment and bone tissue regeneration. In this study, a 3D akermanite (AKT) scaffold was co-loaded with calcium peroxide (CaO_2_) and iron oxide (Fe_3_O_4_) nanoparticles for synergistic magnetic hyperthermia, a Fenton-like reaction and the H_2_O_2_ self-sufficient nanocatalytic effect. The calcium peroxide nanoparticles provided sufficient amounts of H_2_O_2_ for the ferric ions to react through a Fenton-like (Fe^3+^/H_2_O_2_) reaction and also offered Ca2+ ions to support bone regeneration. In addition to being the main source for the Fenton-like reaction, the iron oxide nanoparticles catalyzed the ROS formation and promoted antitumor effects through magnetic hyperthermia. The 3D scaffolds significantly inhibited tumor growth in vivo in MNNG/HOS osteosarcoma tumor-bearing mice with no obvious tumor recurrence. Additionally, the 3D scaffolds showed elevated protein adsorption and biodegradability, releasing Ca2+ ions that are significantly important for promoting biological interactions with the surrounding tissues and further stimulating osteogenesis in rBMSCs (rat bone marrow stem cells), as evidenced by the expression levels of ALP and the expression of osteogenic genes (BMP2, OCN, RUNX2, and COL1). The osteogenesis capacity of the implanted 3D scaffolds was further assessed in vitro in calvaria defect models of SD rats by analyzing bone volume/tissue volume (BV/TV), the trabecular number (Tb.N) and trabecular thickness (Tb.Th), resulting in enhanced bone-regeneration. In another study by Lu et al. [84], porous 3D TTE scaffolds of mesoporous bioglass (MB)/chitosan (CS) (MBCS) were modified with strontium hexaferrite (SrFe12O19) magnetic nanoparticles for bifunctional antitumor effects and bone regeneration. The porous 3D scaffolds promoted cellular attachment and proliferation and further stimulated the osteogenic differentiation of human bone marrow stem cells (hBMSCs), as evidenced by the expression levels of osteogenic-related genes (OCN, COL1, Runx2 and ALP). The new bone regeneration was regulated through the BMP-2/Smad/Runx2 signaling pathway. The in vivo evaluation of the regenerative ability of the 3D scaffolds was assessed in calvaria defect rat models, resulting in new bone tissue formation and mineralization, as confirmed by the bone volume/tissue volume (BV/TV) analysis. The photothermal therapeutic effect of the 3D scaffolds was evaluated under the effect of an NIR laser in osteosarcoma-derived MNNG tumor-bearing rats. The implanted 3D scaffolds inhibited tumor growth and triggered tumor apoptosis and necrosis via the hyperthermia effect.

Bone TTE scaffolds have been significantly researched for secondary or metastatic bone tumors, since the bone tissue microenvironment represents an attractive milieu for the attachment of circulating tumor cells. The invasive tumor cells that escape from the primary tumor site, through lymphatic or bloodstream circulation, are able to localize and colonize bone tissues leading to ECM–cell and cell–cell interactions with osteoblasts and osteoclasts. Thus, bone tumor metastasis develops and is associated with bone inflammations, osteoporosis and bone defects and destruction [33]. The application of multifunctional TTE scaffolds has been studied due to this highly variant microenvironment. Dang et al. [85] developed bioceramic scaffolds made of a poly(d,l-lactide)–β-tricalcium phosphate matrix that were modified with LaB6 micro-nanoparticles. The scaffolds were evaluated for their bone tumor suppression and bone regeneration applications, exhibiting an enhanced mechanical strength and excellent photothermal effects due to the LaB6 modification. The biological properties were assessed in Saos-2 osteosarcoma cell lines, revealing increased tumor cell death under the NIR photothermal effect. Moreover, the release of bioactive La3+, BO3− ions promoted the in vitro osteogenesis of rBMSCs rabbit bone marrow stromal cells as evidenced by the expression levels of the BMP2, RUNX2 and COL 1 osteogenic genes, and further supported their attachment and proliferation. The effective inhibition of bone tumors and the bone regeneration effects were also assessed in vivo and visualized by micro-CT tomography analysis.

In another study by Liao et al. [86], the postoperative recurrence and metastasis of osteosarcoma bone tumors was evaluated using bifunctional hybrid hydrogels composed of methacrylated gelatin/methacrylated chondroitin sulfate in which gold nanorods (GNRs) and nano-hydroxyapatite (nHA) were entrapped. The hydrogel scaffolds were studied in a mouse bone tumor K7M2wt cell line, revealing effective cell death under the photothermal effect. Moreover, the evaluation of the biomimetic properties of the hydrogel scaffolds showed the promotion of the proliferation and osteogenic differentiation of mesenchymal stem cells. The in vivo application in mice models of tibia osteosarcoma depicted the dual functionality of the hydrogel scaffolds in preventing tumor recurrence and in bone regeneration. In a study by Ma et al. [87], bioceramic chitosan TTE scaffolds were developed and doped with nano-hydroxyapatite and graphene oxide composite nanoparticles. The scaffolds were studied for their tumor suppression and bone regeneration effects on human osteosarcoma cells (HOS), pre-osteoblastic MC3T3-E1 cells and hBMSC under photothermal effects with NIR irradiation. The multifunctional scaffolds promoted human osteosarcoma cell death and upregulated the BMP2/Smad signaling pathway to promote osteogenic differentiation in vitro in hBMSCs, under NIR irradiation. Moreover, they expressed good hemostatic effects, and facilitated soft tissue repair, as was indicated by the decreased presence of inflammatory cells and the increased number of collagen fibers. The scaffolds also showed enhanced bone regeneration in vivo as assessed by the post-operative bone volume/tissue volume (BV/TV) ratio through micro-CT analysis.

A multifunctional 3D scaffold was developed by Wang et al. [88] for bone tumor treatment, regeneration and the prevention of recurrence. The 3D-printed scaffold was composed of a hierarchically porous and mechanically strong poly(lactic-co-glycolic acid) matrix containing β-tricalcium phosphate (β-TCP) nanoparticles, and it was further loaded with 2D black phosphorus (BP) nanosheets, doxorubicin (DOX) and osteogenic peptide. The biomimetic and mechanical properties of the 3D nanocomposite scaffolds were comparable to the human cancellous bone. The bone regeneration ability was evaluated in vitro on rBMSCs which showed increased proliferation levels and enhanced adhesion within the porous matrix. The maturation of the rBMSCs and the in situ delivery of the osteogenic peptide further improved rBMSCs osteogenic differentiation and mineralization. Moreover, upregulation of the osteogenic gene expression levels (RUNX2, ALP and COL I) of rBMSCs was observed. The in vivo bone regeneration was evaluated in cranial defects in rat models in which the 3D scaffolds were implanted. The bone volume/tissue volume (BV/TV) ratio and the bone mineral density were assessed confirming that the regenerated new bone tissue had a sandwich-like structure, wherein capillary vessels could be observed. The in vitro and in vivo inhibition of tumor growth was evaluated under synergistic photothermal and chemotherapy effects. The cellular death of osteosarcoma MG63 cells was promoted under an NIR laser and DOX release. Tumor growth was significantly suppressed, and even eliminated, in tumor-bearing nude mice, and the tumor recurrence rate was reduced due to the sustained and localized release of DOX.

**Table 2:** Applications of 3D tumor engineering scaffolds in bone cancer.

Carrier Type	Agent	Characteristics	Ref.
α-tricalcium phosphate/calcium sulfate cements/Fe_3_O_4_/GO	Hyperthermia	Promoted bone regeneration in rat bone marrow-derived mesenchymal stem cells and in cranial defect models of rats; effective tumor inhibition in osteosarcoma and lung metastasis tumor-bearing mice.	[77]
3D scaffolds of gelatin nanofibrous/ZIF8 nanoparticles	Phenamil, photothermal effect	Promoted bone morphogenic protein 2, induced osteogenic differentiation under NIR, alkaline phosphatase activity, increased expression of Col, RUNX2, BSP bone-related genes and C2C12 myoblast cells, osteosarcoma cell death, inhibited tumor growth.	[78]
3D scaffolds gelatin/akermanite/multiwalled carbon nanotubes/magnetic nanoparticles	No agent	Adequate biodegradation rate, increased protein adsorption rate and good biocompatibility in osteoblast G292 cells.	[81]
porous calcium-zirconia scaffolds/magnetic nanoparticles/chitosan	No agent	Biocompatible with satisfactory mechanical and physical properties, no toxicity effects were reported upon treatment with bone marrow stem cells, increased proliferation rate and bone regeneration ability.	[82]
3D akermanite (AKT) scaffold/CaO_2_ and Fe_3_O_4_ nanoparticles	Hyperthermia/Fenton-like reaction	Catalyzed ROS formation, promoted antitumor effects, elevated protein adsorption, biodegradability, stimulating osteogenesis in rBMSCs, increased alkaline phosphatase and osteogenic genes (BMP2, OCN, RUNX2, and COL1) expression, enhanced bone-regeneration.	[83]
3D scaffolds of mesoporous bioglass/chitosan/strontium hexaferrite	Photothermal effect/NIR laser	Promoted attachment, proliferation, osteogenic differentiation of human bone marrow stem cells, increased expression levels of osteogenic-related genes (OCN, COL1, Runx2 and ALP), bone regeneration through the BMP-2/Smad/Runx2 signaling pathway, inhibited tumor growth and triggered apoptosis and necrosis.	[84]
inhibited tumor growth and triggered tumor apoptosis and necrosis/LaB_6_ micro-nanoparticles	NIR photothermal effect	Enhanced mechanical strength, excellent photothermal effect, osteogenesis of rBMSCs rabbit bone marrow stromal cells, increased expression levels of BMP2, RUNX2 and COL 1 osteogenic genes, inhibition of bone tumor and bone regeneration effects.	[85]
3D hydrogels methacrylated gelatin/chondroitin sulfate/gold nanorods/nHA	Photothermal effect	Promotion of proliferation and osteogenic differentiation of mesenchymal stem cells, prevention of tumor recurrence and in bone regeneration.	[86]
3D bioceramic chitosan scaffold/nHA/GO	Photothermal effect/NIR laser	Promoted human osteosarcoma cell death, upregulated the BMP2/Smad signaling, promoted osteogenic differentiation, enhanced bone regeneration.	[87]
3D printed scaffold/β-tricalcium phosphate/2D black phosphorus/osteogenic peptide	Doxorubicin	Biomimetic and mechanical properties comparable to the human cancellous bone, bone regeneration ability, increased osteogenic gene expression levels (RUNX2, ALP and COL I), increased bone mineral density and capillary vessels formation.	[88]

### 2.3. Other Cancer Types

Tumor tissue engineering has found cutting edge applications in the study of metastatic cancer development and progression. The design and formulation of 3D TTE scaffolds that mimic the biological and mechanical features of the tumor microenvironment has been advantageous for the investigation of the metastatic potential of various tumor types (Table 3). Prostate cancer (PCa) at an advanced grade is highly resistant to androgen-deprived therapy (ADT), which is a first-line treatment. The increased drug resistance is the outcome of the upregulated activation of the androgen signaling pathway, resulting in the increased metastatic potential of prostate cancer cells. The bone marrow milieu has been a favorable microenvironment for PCa metastasis that is mediated by the cell–cell interactions between prostate cancer cells and bone endothelial cells. The migrating PCa cells that have followed epithelial-to-mesenchymal transition (EMT) can promote bone tissue colonization leading to osteoblasts lesions [89]. The 3D TTE scaffolds offer a biomimetic matrix for cellular adhesion, colonization, invasion and progression in distant sites.

In a study by Fitzgerald et al. [90], 3D collagen-based bioceramic scaffolds were evaluated as ECM models for cell culture studies of prostate-derived bone tumor metastasis. The PC3 and LNCaP prostate tumor cell lines were cultured in the 3D scaffolds and were allowed to grow and secretions of matrix metalloproteinase (MMP) and prostate-specific antigen (PSA) were allowed to proliferate. Docetaxel and siRNA were transferred to the 3D scaffolds through cyclodextrin-based nanoparticles for effective gene silencing. The PC3 cells grown in the 3D scaffolds expressed reduced levels of MMP1 and MMP9, while the LNCaP cells secreted increased levels of PSA. Moreover, increased drug resistance was expressed in both cell lines, while siRNA resulted in suppression of endogenous GAPDH gene expression. Bone tumor ablation, such as osteosarcoma, is related to bone defects, soft tissue injury and a high chance of recurrence. In another study by Wang et al. [91], chitosan–alginate 3D porous scaffolds were developed as a platform for prostate cancer tumor spheroids, in order to mimic the in vivo responses to targeted gene delivery. The 3D scaffolds were loaded with cationic nanoparticles for plasmid DNA gene and chlorotoxin (CCTX) delivery. Mouse TRAMP-C2 (TC-2) PCa cells were allowed to attach to and proliferate within the 3D scaffold forming malignant tumor-like spheroids with increased expression levels of ECM-related (COL1A1, LAMININ A5) and EMT-related (SNAIL, SLUG, TWIST, SIP1) genes. The expression level of the mRNA epithelial marker E-cadherin was upregulated, indicating that the PCa cells are in a stem-like state that is associated with increased migratory potential. The presence of CTX-targeted nanoparticles resulted in an elevated penetration capacity suggesting their intra-spheroid distribution through receptor-mediated endocytosis, due to the presence of MMP-2 cellular receptors. Moreover, significant targeted gene delivery was demonstrated by the 3D scaffolds that were comparable with the in vivo syngeneic mouse models.

In a recent study by Dozzo et al. [92], 3D scaffolds of nano-hydroxyapatite (nHA)/PLGA were developed to evaluate the effect of scaffold composition on the metastatic potential of prostate cancer cells in the bone. The presence of nHA in the 3D scaffolds highly affected the mechanical properties resulting in weaker structures and degradation behavior promoting cell viability. The multicellular attachment of human osteoblastic hFOB 1.19 and metastatic PC-3 prostate cancer cells was promoted within the 3D scaffold in order to assess gene expression levels (ALP, COL1A1, COL4A1, OPN), proliferation and differentiation behavior. The increasing presence of nHA resulted in the downregulation of gene expression levels, probably due to changes in the micro-pH experienced by the cells. The lower micro-pH probably resulted in the increased proliferation rate of the PC-3 cells in contrast to the osteoblastic hFOB 1.19. Moreover, the 3D scaffolds were used in the drug screening of docetaxel so that a significant reduction in cell viability was demonstrated in the 2D culture models and in the 3D co-cultures. However, the 3D mono-cultures of hFOB 1.19 or PC-3 were significantly less responsive to docetaxel treatment.

Metastatic tumor cells are affected by mechanical extracellular factors within the bone microenvironment, including matrix stiffness through stromal activation, interstitial fluid pressure, constant pressure owing to tumor growth, cell–cell and cell–ECM interactions. Ditto et al. [93], studied the effect of extracellular bone-like mechanical stimuli on the metastatic potential of bone metastasis prostatic adenocarcinoma PC-3 cells that were embedded in a 3D collagen type I scaffold. Mechanical strain was applied to the 3D scaffold through an EQUicycler system in order to study the effect of the mechanical stimuli on cytoskeletal organization and the proliferation and invasion of PC-3 cells. Differential morphological changes and polarized cell elongation were observed on the PC-3 cells depending on the mechanical loading conditions, indicating the stimulation of cytoskeletal reorganization associated with increased metastatic ability and invasion of the tumor cells within the 3D scaffold. In another study by Xu et al. [94], 3D scaffolds composed of chitosan–alginate with tunable stiffness were developed for their application as mimetic platforms for different grades of prostate cancer in order to study prostate metastatic progression. The 3D scaffolds were cultured with non-functional androgen receptor (AD) PC-3 prostate cancer cell lines, bone metastasis-derived C4-2B cell lines with functional AR and human prostatic carcinoma xenograft 22Rv1 cell lines. These cell lines provided osteolytic (PC-3) and osteoblastic (C4-2B, 22Rv1) phenotypes that are observed in human patients. The 3D scaffolds resulted in the formation of multicellular spheroids of increased seeding efficacy and stiffness independent of growth, with necrotic regions or cell cycle arrest being formed within the spheroids. The PCa phenotype was expressed in the 3D scaffolds as evidenced by the expression levels of related biomarkers (pEGFR, AR and cytokeratin 8 (KRT8)). However, different PCa phenotypes were expressed depending on the cell line. The C4-2B and 22Rv osteoblastic prostate cancer cell lines mineralized in the basal media, in contrast to PC-3. Moreover, varied expression profiles of PCa-related genes (PSA mRNA, LIMK1 mRNA) and proteins (AR) were demonstrated by the different cellular lines in the 3D scaffolds.

Long et al. [95] studied sphere-templated poly(2-hydroxyethyl methacrylate) (pHEMA) hydrogel as 3D scaffolds for human PCa cell lines. The developed scaffolds were used as engineering xenografts for implantation in athymic nude mice in order to evaluate their ability to generate tumors in vivo. The 3D scaffolds were pre-cultured with M12 tumorigenic cells, resulting in the development of xenografts in vivo, after implantation. The scaffolds presented a necrotic core with viable tumor tissue in the surrounding area, and tumor cells had proliferated on the outside the scaffold area invading the nearby tissues. In the case of LNCaP C4-2 cells, the implanted scaffolds were poorly tumorigenic, revealing the absence of pro-tumorigenic signaling. The 3D scaffolds with non-tumorigenic M12mac25 cells attached expressed a tumorigenic response with significant F4/80+ macrophage infiltration being present in close proximity to the M12mac25 tumor clusters within the scaffolds. The tumor growth in the case of M12mac25 cells was probably attributed to the cell signaling mediated by the presence of tumor-associated macrophages that can activate pro-tumorigenic signaling pathways [96,97].

The important interactions between prostate cancer-associated fibroblasts (CAFs) and the ECM in prostate cancer progression were investigated by Pereira et al. [98]. Human PCa microtissue models were developed by primary patient-derived CAFs that were attached in 3D poly(ε-caprolactone) scaffolds and allowed to proliferate. The 3D scaffolds provided spatial support for CAFs’ growth and the deposition of extensive native ECM, as evidenced by the increased levels of fibronectin and collagen IV. In this way, modular stromal microtissue models were created and non-malignant BPH-1 human benign prostate epithelial cells were added to assess the morphological changes promoted by CAFs in prostate epithelia. It was demonstrated that CAFs promoted the morphological transition of benign epithelia, since within CAF microtissues, the BPH-1 cells were elongated, with larger size, and were highly oriented with the underlying stroma. Moreover, tryptase-positive mast cells were added to the CAFs microtissues since they modulate epithelial–stroma interactions and promote tumorigenesis. The incorporation of HMC-1 mast cells promoted the morphometric transition and malignant phenotype of benign epithelia via a tryptase-mediated mechanism. In this study, a novel mechanism of mast cell–stroma crosstalk in the prostatic microenvironment was indicated.

In addition to PCa, TTE 3D scaffolds have also been applied in the study of cancer pathogenesis and the microenvironment of various cancer types, including liver, pancreas and colon cancer. In the case of liver cancer, and especially that of hepatocellular carcinoma (HCC), an extensive review on the recent progress has been presented by Shao et al. [99]. In HCC liver cancer, 3D scaffolds have mostly been applied for the development of 3D hepatoma spheroids and organoids for drug screening and personalized medicine through patient-derived tumor xenografts in order to mimic the parental tumor microenvironment and HCC heterogeneity. Hydrogel scaffolds, microfluidic devices and organ-on-a-chip are only a few examples of strategies used for evaluating HCC development and bone metastasis [100]. Tissue engineering applications in the pancreas are an emerging research field, especially in the investigation of the biochemical and biomechanical interactions between pancreatic ductal adenocarcinoma (PDAC) leading to the ECM taking on a desmoplastic and fibrotic structure.

In a study by Gupta et al. [101], a polyurethane (PU)-based 3D scaffold was created as a multi-cellular model for PDAC development. The 3D scaffold served as the carrier of three cellular cultures, including PANC-1 pancreatic cancer cells, HMECs human microvascular endothelial cells and PS-1 pancreatic stellate cells. For the effective attachment and proliferation of the tri-cultured cells, the 3D PU scaffold was designed with tunable porosity and an elastic modulus, while the stiffness was similar to the PDAC ex vivo tissues. Moreover, the 3D scaffold was divided into two areas, an inner that was surface functionalized with fibronectin (FN) and a surrounding that was coated with collagen (COL) type I. The two distinct areas were developed in order to create optimal growth conditions for the PANC-1 cancer cells (inner area) and the stromal compartments consisting of the PS-1 stellate and HMECs endothelial cells (surrounding area). The protein coating of the 3D scaffolds resulted in differential cellular interactions and growth depending on the cell types being co-cultured. PANC-1 cells were mainly located in the FN-rich area, with the HMEC endothelial cells being located in the COL-rich area, and the PS-1 stellate cells being localized in both FN- and COL-rich areas. This way, the growth and progression of a PDAC-mimicking microenvironment was supported by the varied cell types. The tri-cultured 3D scaffold promoted elevated collagen production, even in the FN-rich inner area, mimicking PDAC desmoplasia. Moreover, the stellate cells expressed a fibril alignment, as evidenced by the αSMA expression levels, which was attributed to activated stellate cells, while CD31+ HMEC endothelial cells were observed within the stellate fibrous stroma. The PANC-1 cells consisted of heterogeneous cellular populations since the upregulation of pan-Cytokeratin and CD-24 biomarkers were expressed. All three cell types expressed an ability to migrate between the two zones of the hybrid scaffold.

In another study by de la Pena et al. [102], a self-assembling hydrogel approach to peptide amphiphiles (PAs) was followed for the development of 3D scaffolds with multiple ECM components of PDAC, including collagen type I, fibronectin, laminin and hyaluronan. The 3D scaffolds presented tunable fibrillary structures, thicknesses and stiffness depending on the composition of the various components. Patient-derived pancreatic stellate cells (PSCs) and primary macrophages were co-cultured in the 3D hydrogel scaffolds in order to mimic the PDAC stroma cellular components. Moreover, patient-derived PDAC cells were used in order to produce a triple co-cultured cellular system. The 3D co-cultures of PDAC and stromal cells generated duct-like organoid colonies with extensive stroma mimicking the topology of PDAC ECM. Moreover, the 3D cultured scaffolds maintained patient-specific transcriptional profiles as expressed by the SOX2 and KLF4 transcriptional factors, and exhibited the functionality of cancer stem cells (CSC) (CD133 +/CXCR4+ PDAC cells) as evidenced by the gene expression involved in oxidative phosphorylation. Depending on the composition of the 3D PA scaffolds it was possible to modify the expression of niche-dependent genes, such as ECM receptors (ITGB1, CD44s, CD44v6) and regulators (MMP14, LOXL2). The patient-derived 3D models enabled epithelial-to-mesenchymal transition, as expressed by ZEB1 and NANOG gene expression, and matrix deposition. The PDAC 3D models described [101,102] represent important platforms for drug screening and personalized medicine applications.

Colorectal cancer (CRC) is another growing research field for TTE 3D scaffolds with the aim of replacing the tumor ECM in order to investigate drug screening, biomechanical and biochemical properties, cellular interactions, tumor progression and metastasis. These 3D scaffolds aim to provide assistance to our understanding of CRC progression and support alternative methods for developing new therapeutic strategies. The advancements in bioengineered 3D scaffolds for use in drug screening for CRC have been presented by Sensi et al. [103] in a recent review article. The 3D models find applications in mimicking the avascular compartments of the tumor ECM in terms of biochemical and biomechanical stimuli, tissue oxygenation and nutrient supply. Their development is applied in the investigation of CRC tumor heterogeneity, tumor–host interactions, our understanding of tumor biology and the evaluation of novel therapeutic agents and personalized medicine [103].

In a study by Chen et al. [104], 3D-printed scaffolds made of polycaprolactone were developed and modified with collagen type I. Tumor-associated stromal cells were allowed to attach to and proliferate in the 3D scaffold to form a 3D in vitro tumor model. The developed 3D tumor model was co-cultured with cancer-associated fibroblasts (CAFs) and tumor-associated endothelial cells (TECs) in order to provide a scaffold that could mimic the cell–cell and cell–ECM physiological functions. For this reason, normal stromal cells (HELFs and HUVECs) were activated and transformed into CAFs and TECs, respectively. The spatial structure of the 3D scaffolds supported the physical attachment of the cells and promoted niche tumor development including the CAFs and TECs. The activated stromal cells overexpressed tumor-associated biomarkers, such as MMP2 which is secreted by fibroblasts and proliferation-related protein Ki67 which represents a critical biomarker of CRC. The expression of MMP2 and Ki67 was indicative of the promoted ECM remodeling and increased proliferation rate of the cells in the 3D scaffolds. The reprogramming of stromal cells to a malignant phenotype was evidenced by the increased expression levels of CAF markers, such as α-SMA, FAP and FSP-1. Moreover, the expression levels of genes encoding tenascin C (TNC), collagen I and TGF-β1 were upregulated, signifying paracrine signals from CAFs and an activated fibroblast phenotype. The upregulation of metabolic signals related to hypoxia, stress and anti-apoptosis, such as HIF-1, MAPK and ErbB, respectively, was accompanied by the downregulation of necrosis (TNF) factor and inflammation (IL17) metabolic signals. These findings indicated that the in vitro 3D tumor tissues within the scaffolds were in highly relevant to the physiological functions of the EMT/Wnt signaling pathways involved in CRC in vivo tumor tissues.

Moreover, 3D TTE has found interesting applications in the study of the fundamental cellular niche and mechanical properties associated with CRC metastasis. The widespread understanding of the mechanism related to CRC metastatic potential has been reviewed by Sarvestani et al. [105], who presented 3D ex vivo models of biomaterials and advances in organ- or body-on-a-chip techniques. In a study by D’Angelo et al. [106], patient-derived decellularized ECM scaffolds were used, originating from a healthy liver (HL), colorectal cancer liver metastasis (CRLM) and CRC and a healthy colon (HC). The use of decellularized scaffolds was preferred since the 3D models retained the phenotype of the tissue of origin. HT-29 cells were attached to and proliferated in the CRC, HL and CRLM decellularized scaffolds promoting the formation of clusters of different sizes. The expression levels of proliferation-related protein Ki67 and Caspace-3 were higher in CRC scaffolds with no significant difference in liver scaffolds. The migration ability of HT-29 cells was significantly higher in CRLM and CRC scaffolds mainly due to the increased presence of reticular collagen fibers compared to the healthy tissue scaffolds (HL and HC). The recellularization of the CRLM scaffolds and EMT displayed by HT-29 cells were attributed to the reduced E-cadherin expression, accompanied with the overexpression of vimentin. These 3D patient-derived models recapitulated the metastatic microenvironment to a high degree, as evidenced by the gene expression profiling involved in demethylation, deacetylation, metabolic stress and hypoxia.

**Table 3:** Applications of 3D tumor engineering scaffolds in varied tumor types.

Carrier Type	Agent	Characteristics	Ref.
3D collagen-based bioceramic scaffolds/PC3, LNCaP prostate cells	Docetaxel and siRNA	Secrets matrix metalloproteinase and prostate-specific antigen (PSA), expressed reduced levels of MMP1 and MMP9, LNCaP cells secreted increased levels of PSA, suppressed endogenous GAPDH gene expression.	[90]
chitosan–alginate 3D porous scaffolds/prostate cells	plasmid DNA gene/chlorotoxin	Increased expression levels of ECM-related (COL1A1, LAMININ A5) and EMT-related (SNAIL, SLUG, TWIST, SIP1) genes; upregulation of mRNA epithelial marker E-cadherin.	[91]
3D scaffolds of nano-hydroxyapatite (nHA)/PLGA/prostate cells	Docetaxel	Degradation behavior promoting cell viability, increased proliferation rate, significant reduction in cell viability.	[92]
3D collagen scaffold/metastatic prostatic adenocarcinoma	No agent	Stimulation of cytoskeletal reorganization associated with increased metastatic ability and invasion.	[93]
3D scaffolds of chitosan-alginate/prostate metastasis cancer	No agent	Osteolytic (PC-3) and osteoblastic (C4-2B, 22Rv1) phenotypes observed in human patients, formation of multicellular spheroids, expression of pEGFR, AR and cytokeratin 8 biomarkers, mineralization.	[94]
3D polymeric hydrogel/M12 tumorigenic cells/prostate cells	No agent	Tumorigenic response with significant F4/80+ macrophage infiltration.	[95]
3D poly(ε-caprolactone) scaffolds/prostate microtissue models	No agent	Spatial support for CAFs’ growth, morphological transition of benign epithelia, epithelial–stroma interactions and promote tumorigenesis.	[98]
3D hepatoma spheroids and organoids	Drug screening	Mimic the parental tumor microenvironment and HCC heterogeneity.	[99]
polyurethane (PU)-based 3D scaffold/fibronectin/collagen/PDAC	No agent	Optimal growth conditions, stiffness similar to PDAC ex vivo tissues, differential cellular interactions and growth depending on the cell types, activated stellate cells from αSMA expression levels, CD31+ HMEC endothelial cells.	[101]
3D scaffolds/peptide amphiphiles/Patient-derived pancreatic stellate cells	No agent	Duct-like organoid colonies with extensive stroma mimicking the topology of PDAC, SOX2 and KLF4 transcriptional factors, cancer stem cells’ functionality (CD133 +/CXCR4+ PDAC cells).	[102]
3D printed polycaprolactone scaffolds/collagen/stromal cells	No agent	Expression of MMP2 and Ki67 indicative of ECM remodeling, reprogramming of stromal cells, increased expression levels of CAF markers (αSMA, FAP, FSP-1), upregulated metabolic signals related to hypoxia, stress and anti-apoptosis (HIF-1, MAPK ErbB).	[104]
patient-derived decellularized ECM scaffolds/colon HT-29 cells	No agent	Reduced E-cadherin expression, overexpression of vimentin, recapitulation of metastatic microenvironment, demethylation, deacetylation, metabolic stress and hypoxia genes expression profiling.	[106]

## 3. Tissue Engineering in Cancer Therapeutics: Immunotherapy

**Figure 2:**
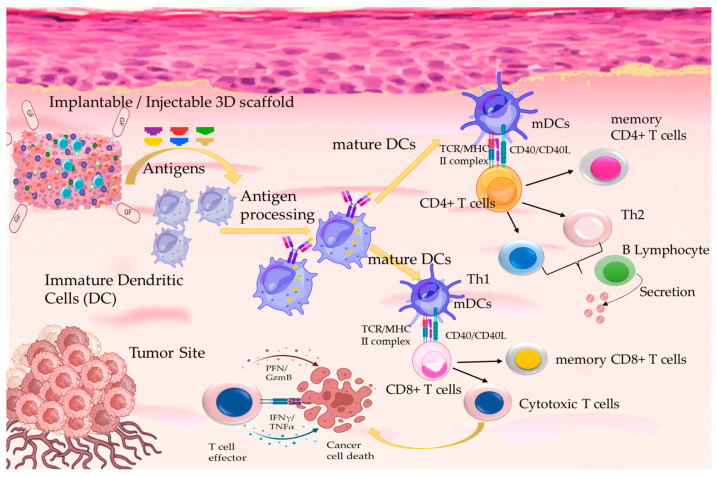
Schematic representation on the effect of 3D TTE scaffolds with antigen-specific immunotherapies. (Created with the assistance of BioRender.com https://app.biorender.com/user/signin?illustrationId=6156d45891063d00af8af51d (accessed on 27 April 2024 up to 28 April 2024) and Microsoft ppt (software version number: Microsoft Office Professional Plus 2016)).

The applications of 3D TTE scaffolds in cancer immunotherapy have drawn extensive attention, aiming to advance the understanding of the mechanisms and immunological processes involved in escaping immune surveillance and the initiation of host immunity in order to eliminate malignancies and restrain cancer metastasis and recurrence. Immunotherapies represent a central backbone for cancer treatment, enabled by the advancements in genomics and proteomics that resulted in the identification of patient-specific mutations and biomarkers, further promoting the progress of personalized immunotherapies. The main immunotherapeutic approaches that have been applied in 3D TTE include blocking antibodies or inhibitors specifically for suppressing immune checkpoint pathways, such as cytotoxic T lymphocyte-associated antigen-4 (CTLA-4) and programmed cell death receptor-1 (PD-1) [107]. Moreover, cellular therapies utilizing patient-derived immune cells, such as dendritic cells (DC) and engineered T cells, have been introduced into cell-based 3D TTE scaffolds in order to study the cellular and cell–matrix mechanisms that contribute to TME pathophysiology in a more personalized manner [26]. Immune vaccines and cancer viruses have been utilized in 3D TTE in order to understand the underlying mechanisms of neoantigen-induced antitumor immune responses, so as to design new neoantigen-based immunotherapies [108]. To promote effective immunotherapies, the challenges posed by the design requirements of the 3D scaffolds are being considered in terms of biomechanical and biochemical properties to assist in the development of the immunosuppressive TME. These concepts have promoted scientific research of 3D biomaterial scaffolds (Table 4) composed of patient-derived immune cells for the localized delivery of immunotherapies in combination with cell therapies, such as CAR-T cells and nanostructured materials, such as nano-vaccines with neoantigens (Figure 2) [109,110,111].

Cancer immune checkpoint blockade (ICB) therapy was investigated by Yu et al. [112] in an injectable bioresponsive gel scaffold. The 3D hydrogel scaffold was composed of a triblock thermo-responsive copolymer of polyethylene glycol and polypeptides that contained ROS-responsive L methionine and dextro-1-methyl tryptophan (D-1MT), a tryptophan derivative that is able to prevent the suppression of T-cell responses (anergy) triggered by IDO (indoleamine-2,3-dioxygenase). The hydrogel scaffold was loaded with an anti-programmed cell death-ligand 1 (anti-PD-L1) antibody that is able to regulate PD-1. The injectable scaffold served as a platform for localized drug delivery and the modulation of the ROS level in the TME, thus enhancing melanoma treatment in melanoma-bearing female C57BL6 mice. The ROS levels represent important signals in the immune system, which are closely linked with the immunosuppressive TME by inducing apoptosis and regulating PD-1 expression. Thus, the sustained release of anti-PD-L1 antibody and D-1MT in vivo resulted in effective T cell-mediated immune responses through the elevated presence of tumor-infiltrating CD45+ and CD8+ T cells in the TME of collected tumor tissues. In another study by Neal et al. [113], patient-derived organoids (PDOs) from human biopsies of primary and metastatic tumors or mouse tumors containing tightly integrated epithelial and stromal compartments were used for the modeling of TME-intrinsic immune responses. PDOs tumor fragments from common tumor sites, including the colon, pancreas and lung, were embedded in type I collagen matrix. The developed PDOs preserved their tumor architecture and fibroblast stroma. Interestingly, the PDOs contained tumor-infiltrating lymphocytes (TILs), including CD14+ and CD68+, macrophages, T cells, B cells, NK cells and infiltrating CD3+ T cells expressing the immune checkpoint surface receptor programmed cell death protein-1 (PD-1). The PDOs shared diverse immune cell populations and expressed M2 macrophage phenotype that reproduced the TME of the original biopsies. These PDOs were applied for the study of immune checkpoint blockade (ICB) with anti-PD-1 and anti-PD-L1 that regulated the inhibition of PD-1 and PD-L1. Thus, tumor antigen-specific TILs were activated resulting in elevated immune reaction and late apoptotic and necrotic tumor cells. Such applications are critical for personalized immunotherapy evaluation.

In a recent study by He et al. [114], photothermal 3D printed biodegradable scaffolds were combined with checkpoint blockade immunotherapy for the effective treatment of metastatic tumors. For this study, 2D niobium carbide (Nb2C) MXene nanosheets were coated with a mesoporous silica layer for the loading of immune adjuvant R837. The functional nanosheets were further integrated onto 3D-printed biodegradable bioglass scaffolds. The developed 3D scaffolds exhibited a profound photothermal-conversion capacity that effectively promoted photonic hyperthermia under NIR irradiation. Hyperthermia resulted in the reduced viability of breast cancer cells in in vitro co-cultures, and in tumor ablation in 4T1 breast cancer models in BALB/c mice. After tumor ablation, the remaining tumor debris was released from the tumor site to the 3D scaffolds stimulating the vaccine-like effect of R837 which promoted the recruitment and maturation of dendritic cells (DCs), as expressed by the percentage of mature DCs (CD11c+, CD80+, CD86+). The activated immune responses that were promoted by the mature DCs resulted in the stimulation of increased cytokine secretion levels (IL-6, IL-17, TNFα). Moreover, the 3D scaffolds were evaluated in combination with the anti-PD-1/PD-L1 antibody in breast cancer orthotopic distant metastasis (lung and bone) murine models. The combination of photothermal hyperthermia and anti-PD-L1 demonstrated profound inhibitory effects on primary and distant tumors, implying an enhanced suppression of tumor metastasis. The investigation into the underlying mechanisms of antitumor and anti-metastatic effects denoted the pivotal role of cytotoxic T-lymphocytes (CTL), especially CD8+, CD45+ and CD3+T cells. The ratio of CTLs to Treg cells indicates the increased immune activity of CTLs, which can probably be associated with protection against tumor recurrence through immune memory. Furthermore, the 3D scaffolds promoted osteogenesis through the bioglass matrix and the degradation products of Nb- and Si- species, which facilitate bone regeneration.

The concepts of 3D TTE have been applied to cell therapies in order to provide effective scaffolds for the maintenance and specific delivery of cells and drugs to the tumor sites. In a study by Atik et al. [115], 3D hydrogels of low viscosity were developed from hyaluronic acid and gelatin for the maintenance and delivery of tumor-specific chimeric antigen receptor (CAR) T-cells. The direct intracranial infusion of CAR T-cells has been evaluated through CED (convection enhanced delivery) for glioblastoma treatment. CED can effectively deliver small therapeutic agents directly into the intracranial area through a catheter under positive pressure using a slow infusion rate. The application of positive pressure has been related to an improved delivery and distribution of the therapeutic agents. However, in this study the traditional CED infusion of CAR T-cells resulted in an inefficient intratumoral delivery that was associated with cellular sedimentation. The main reason behind CAR T-cells’ sedimentation was the extensive time of delivery for CED infusion (about 4 to 6 h). Thus, CAR T-cells were suspended in the 3D hydrogels that presented a biodegradable and low viscosity matrix that supported the delivery of living cells. The 3D hydrogels prevented CAR T-cells sedimentation, maintained cellular viability and promoted elevated cellular delivery, with no acute toxicity on brain parenchyma associated with intracranial infusion. The EGFRvIII-specific CAR T-cells effectively migrated from the 3D hydrogel by mediating tumor-specific cytotoxicity in U87MG and U87MG.DEGFR glioma cells.

In another study by Yang et al. [116], a 3D hydrogel scaffold was studied to improve the efficacy of immunotherapies in combination with chemotherapy for optimized therapeutic outcomes in B16 melanoma tumor-bearing C57BL/6 mice. For the development of the 3D scaffolds, pH-sensitive nanoparticles of doxorubicin and CpG immune adjuvant in combination with dendritic cells (DCs) were embedded in α-cyclodextrine hydrogels. The pH-sensitive 3D hydrogels induced a controlled DOX release that stimulated the expression of tumor antigens. The DOX concentration was adjusted in order to promote immunogenic cell death (ICD) without affecting DCs viability. The expression levels of high-mobility group protein B1 (HMGB-1) were increased, promoting ICD. The HMGB-1 is a DAMP (damage associated molecular pattern) that is released by damaged or dying cells to activate the innate immune system by interacting with PRRs (pattern recognition receptors). Subsequently, the HMGB-1 expression promoted immunostimulatory effects, exciting immune cells to become antigen-presenting cells, as evidenced by the expression levels of CD86 molecules on CD11c+ cells. DCs are the principal antigen-presenting cells that patrol for DAMPS signals, stimulating their maturation [117]. Thus, increased DC maturation was promoted with significantly elevated immunogenicity. Then, the combined stimulation of DCs, antigens and immune adjuvants in the 3D scaffold provided abundant tumor-associated antigens that were recruited and processed by the endogenous host DCs, while the exogenous DCs initiated adaptive immunity. The underlying mechanism of the combined and synergistic effects of the 3D scaffold in chemo-assisted immunotherapy was based on the upregulation of cytotoxic T-lymphocytes’ (CTLs) tumor effects, as evidenced by the significantly increased expression levels of activated CD4+ and CD8+ T cells. Moreover, the secretion levels of INF-γ and IL-2 signified the elevated infiltration of effector T cells that resulted in the decline of the immunosuppressive TME, thus maximizing the adaptive and innate immune responses.

The important aspects of neoantigen-based cancer vaccines were also studied by Yang et al. [118] through the development of polymeric hydrogel 3D scaffolds of cyclophosphamide (CTX) for amplified cancer immunotherapy. The CTX 3D scaffolds were evaluated in combination with CpG-adjuvant and tumor-lysate loaded 3D hydrogels. The CTX scaffolds were intratumorally administered in BALB/c mice of CT26 colon tumor models, and then the CpG/tumor–lysate scaffolds were subcutaneously administered. The 3D scaffolds promoted the sustained release of CTX that caused immunogenic cell death (ICD) providing a pool of tumor antigens and immunostimulating DAMPs that were recognized by DCs for priming innate immunity. The immune stimulation effects resulted in the maturation and activation of DCs, as evidenced by the increased presence of CD11c+, CD11c+CD86+, CD11c+MHC-I+ DC cells. Moreover, the cytotoxic T-lymphocyte anti-tumor immune effects were verified by the elevated expression of CD3+, CD3+CD4+, and CD3+CD8+ T lymphocytes. The combination of the 3D hydrogel scaffolds (CTX and CpG/tumor-lysate) resulted in significantly advanced immune responses promoting the downregulation of CD4+ T cells and the upregulation of CD8+ T cells. Thus, the ratio of CD8+/CD4+ was increased, stimulating the transition of the T cells to the Th1 phenotype. The combination of the 3D scaffolds promoted an enhanced antitumor effect by significantly inhibiting tumor growth. Furthermore, the elevated presence of effector memory T cells and central memory T cells were indicative of the strong antitumor immune memory responses that were further confirmed by the inhibition of both re-inoculated tumor growth in the cured mice and of distant tumor growth.

Cancer stem cells (CSCs) have also been utilized in 3D TTE in order to investigate the underlying biological mechanisms in a TME-mimicking milieu. In a study by Bassi et al. [119], 3D scaffolds were evaluated for the development of a bone-mimicking TME, focusing on the CSCs osteosarcoma niche. For this study, biomimetic hybrid hydroxyapatite-based 3D scaffolds were prepared either by Mg-doped hydroxyapatite (MgHA) and collagen or by porous hydroxyapatite. Both 3D scaffolds were co-cultured with MG63 and SAOS-2 human osteosarcoma cell lines, and CSCs enrichment was succeeded by sarcospheres formation. The 3D scaffolds were designed to present osteogenic and mineralization properties for bone regeneration, thus mimicking the in vivo phenomena of new bone tissue formation. Moreover, both scaffolds presented nanostructured organization for effective cell adhesion, migration and colonization. The physicochemical (porosity, fiber development) and mechanical features (stiffness) of the 3D scaffolds induced an effective stem phenotype in the 3D sarcospheres with increased protein expression levels. The 3D osteosarcoma cells were successfully enriched with CSCs, in order to reproduce the CSC-tumor niche behavior, as evidenced by the increased mRNA expression level of stem-related genes, such as OCT-4, SOX-2 and NANOG. The spheroidal phenotype of the sarcospheres was maintained both in the pores of the HA 3D scaffold and in the fibers of the HA–collagen 3D scaffold. Furthermore, the two scaffolds significantly upregulated the gene expression levels of NOTC-1, HIF-1α, and IL-6 that are involved in the signaling cascade between CSCs and the tumor stem niche, promoting resistance and metastasis. However, different gene expression profiles were reported for the two different scaffolds indicating the varied biomimetic potential of the biomaterial scaffolds.

In another study by Mencia Castano et al. [120], cell-free 3D scaffolds composed of of collagen–nanohydroxyapatite were applied for the transfection of a miRNA inhibitor (antago-miR-133a) in rMSC (rat mesenchymal stem cells) and in rat-calvarial defects. The 3D scaffolds promoted the localized in vivo antago-miR-133a transfection in host cells of immunocompetent animals post-implantation. The inhibition of the miR-133a gene promoted the upregulation of the RUNX2 osteogenesis transcription factor accompanied by increased gene expression levels of ALP, BMP-2 and OCN (osteocalcin) resulting in bone repair activation. Increased mineralization and calcium deposition was observed throughout the 3D scaffolds, even in the central regions. Moreover, an increased bone volume rate was depicted through accelerated caldaria healing with calcified tissue, and enhanced bridging and thickness, indicating de novo bone formation. The host’s cellular immune responses to the antago-miR-133a-loaded 3D scaffold showed the presence of CD11b+ hematopoietic descendant cells in the defects site, and the increased presence of CD206+ cells was indicative of a pro-remodeling M2-like macrophage population, which further suggested a beneficial effect on bone regeneration.

**Table 4:** Applications of 3D tumor engineering scaffolds in immunotherapies.

Carrier Type	Agent	Characteristics	Ref.
3D hydrogel scaffold polypeptides/L methionine	dextro-1-methyl tryptophan (D-1MT)/anti-programmed cell death-ligand 1 (anti-PD-L1) antibody	Sustained release of anti-PD-L1 antibody and D-1MT, effective T cell mediated immune responses, tumor-infiltrating CD45+ and CD8+ T cells.	[112]
patient-derived organoids	anti-programmed cell death-ligand 1 (anti-PD-L1) antibody	Diverse immune cell populations and expressed M2 macrophage phenotype, inhibition of PD-1 and PD-L1, activated tumor antigen-specific tumor infiltrating lymphocytes (TILs).	[113]
3D printed biodegradable bioglass scaffolds/2D niobium carbide	Photothermal effect/immune adjuvant R837	Profound photothermal-conversion capacity, stimulated vaccine-like effect of R837, recruitment and maturation of dendritic cells (CD11c+, CD80+, CD86+), increased cytokine secretion levels (IL-6, IL-17, TNFα), profound inhibitory anti-PD-L1 effect, increased immune activity, bone regeneration.	[114]
low viscosity hyaluronic acid and gelatin 3D hydrogels	CAR T-cells	Prevented CAR T-cells sedimentation, maintained cellular viability, and promoted elevated cellular delivery, tumor specific cytotoxicity in U87MG and U87MG.DEGFR glioma cells.	[115]
3D α-cyclodextrine hydrogel scaffold	doxorubicin and CpG immune adjuvant	Promote immunogenic cell death, increased levels of high mobility group protein B1, promoted immunostimulatory effects, expression levels of CD86 molecules on CD11c+ cells, upregulation of cytotoxic T-lymphocytes.	[116]
polymeric hydrogel 3D scaffolds	cyclophosphamide (CTX)	Immunogenic cell death, maturation and activation of DCs (CD11c+, CD11c+CD86+, CD11c+MHC-I+), cytotoxic T-lymphocyte anti-tumor immune effects (CD3+, CD3+CD4+ and CD3+CD8+), increased stimulating the transition of T cells to Th1 phenotype.	[118]
biomimetic hybrid hydroxyapatite-based 3D scaffolds	Cancer stem cells (CSCs) enrichment	Sarcospheres formation, osteogenic and mineralization properties for bone regeneration, effective cell adhesion, migration and colonization, increased mRNA expression level of stem-related genes (OCT-4, SOX-2, NANOG), upregulated the gene expression levels (NOTC-1, HIF-1α, IL-6), signaling cascade between CSCs and tumor stem niche.	[119]
scaffolds of collagen-nanohydroxyapatite	miRNA inhibitor (antago-miR-133a)	Localized in vivo antago-miR-133a transfection, increased genes expression levels of ALP, BMP-2, OCN, increased bone defect healing, increased presence of CD206+ cells, pro-remodeling M2-like macrophage population.	[120]

## 4. Conclusions

The application of nanostructured biomaterials in tumor tissue engineering with the potential for cancer therapy and regeneration has expanded the number of approaches for understanding the biochemical and biomechanical interactions within tumor microenvironment. A great obstacle to conventional cancer treatments is the remaining tumor cells that possess an increased potential to invade and create metastatic lesions on other healthy tissues, leading to tumor recurrence. The use of novel gene therapies and engineered cell therapies in combination with antigens and nanovaccines has opened up new directions for targeted immunotherapy to overcome the limitations and obstacles of conventional treatments. This review highlights some interesting research approaches to 3D TTE scaffolds that combine nanostructured biomaterials and bioactive agents with cellular adhesion and tissue growth and regeneration in order to mimic the tumor microenvironment. This emerging field of biomimetic 3D scaffolds has increased considerably during the past decade offering new opportunities for understanding the TME and tumor treatment. The driving force is the huge flexibility in terms of the biomaterials employed, the 3D methods used for creating the scaffolds, the co-culturing conditions for tumors and immune cells of varied phenotypes and the simultaneous loading of drugs, growth factors and immune-activating factors. Such scaffolds can facilitate the development of tissue-engineered models, to study the biology of tumor metastasis by evaluating the cell–ECM, cell–host cell and cell–tumor cell interactions. In addition, TTE scaffolds find interesting applications in the complex mechanisms of immunotherapies due to their ability to entrap immune cells including stem cells, dendritic cells, CAR T-cells and antigens. Biomaterials are used to recruit immature antigen-presenting cells, such as DCs, to further promote T-cell immune responses. The significant role of stem cells is due to their self-renewal ability and capacity to proliferate and differentiate to multiple cell types, modulating the host immune system. In this review, the application of nanostructured biomaterials in biomimetic 3D scaffolds is presented in order to study TME physiology and immune responses for effective tumor regression, the inhibition of recurrence and the design of targeted immunotherapies.

## 5. Future Directions

Today, the development of TTE scaffolds plays a significant part in tumor therapy and regeneration due to their multifunctional role as biomimetic matrices that can simulate the TME, the metastasis sites and the complex tissue constructs. Thus, 3D TTE scaffolds offer realistic models to study the biology, physiology and immunology of tumors and the interactions with the surrounding healthy tissues. These areas, in combination with the development of nanostructured biomaterials, nanomedicines and gene therapies, present an optimistic alternative for the evolution of tumor understanding and immunotherapies. However, great challenges remain regarding the application of TTE methods in clinical practice and the patients.

One of the main challenges in tumor tissue engineering is creating 3D scaffolds that have the right mechanical and biological properties to support drug delivery and tissue regeneration, while also providing a suitable environment for cell and tissue growth. Moreover, vascularization of tissue constructs is crucial for the survival and integration of newly formed tissues. Thus, developing biomaterial scaffolds that can promote blood vessel growth and support the formation of a vascular network within large tissue constructs is still a great challenge. The levels of biofunctionality of the biomaterials is a critical parameter, since biomaterials can create nanoscale structures, and deliver biofactors, and gene signals from the extracellular environment. Such biomaterials are evolving as interesting candidates for TTE 3D scaffolds. However, optimizing the technology and improving the clinical safety of biomaterials are still challenges that need to be addressed. Moreover, limitations such as immune rejection, low biological activity and limited availability are still challenging factors that need further research. Thus, the development of new biomaterials that can overcome these limitations and provide better biocompatibility, biodegradability and immune responses are crucial for the future of TTE regeneration and immunotherapies. Also, genetic engineering technology has shown potential in promoting the expression of genes, antigens and growth factors that stimulate innate and adaptive immunity for supporting antitumor effects and even regeneration [33,36]. However, optimizing the delivery of genes and growth factors while ensuring their maintenance and efficient expression in the target cells is still challenging. Furthermore, there are challenges related to optimizing the TTE technology in order to improve clinical safety issues. These challenges include minimizing the risk of infection, increasing host tolerance for reducing the risk of immune rejection, addressing ethical and moral issues for their implementation, and evaluating the long-term potential tumorigenesis risk of cell therapies. Other potential complications are associated with repeatability and scalability of the protocols, and cost issues related to tumor therapy, immunotherapy and tissue regeneration [31].

The future of TTE regeneration and immunotherapies for cancer treatment holds immense promise and potential for transforming the landscape of medicine. Many challenges need to be addressed, but also many opportunities are arising from the combination of 3D scaffolds, biomaterials, nano-delivery and immunotherapies. A great amount of promise is presented by the recruitment of stem cell therapies to the 3D TTE, offering the potential to differentiate into various cell types and promote regeneration. Stem cell-based vaccine therapy offers the opportunity to use a whole cell as a vaccine agent, eliminating the immunosuppressive TME by inducing amplified immune responses through the activation of CD8+ T-cells [121,122]. The embedment of immune checkpoint inhibitors can act synergistically, further elevating host immune responses by the recruitment of effector T-cells. Advancements in cancer vaccine technology include neoantigen vaccines and mRNA-based vaccines, which hold promise for stimulating the immune system to recognize and attack cancer cells [123]. Next-generation TTE immunotherapies should target 3D bioprinted scaffolds that allow the creation of complex 3D tissue structures by using bioinks that incorporate living cells, such as stem cells, immune cells and engineered CAR T-cells, growth factors, immune inhibitors and antigens [124]. The development of such scaffolds broadens the applicability to varied tumor types, while simultaneously promoting regeneration and anplifying immunotherapies. Overall, the future of tissue regeneration and immunotherapies for cancer is bright, with ongoing research and innovation poised to revolutionize treatment options and improve patient outcomes.

## Figures and Tables

**Table 1:** Applications of 3D tumor engineering scaffolds in breast cancer.

Carrier Type	Agent	Characteristics	Ref.
patient-derived xenograft (PDX)	Paclitaxel, Doxorubicin, 5-Fluorouracil	Drug screening in multicellular spheroids, expressed drug resistance, hypoxia, elevated cleaved-PARP expression levels, protection of the tumor cells from drug-induced apoptosis, similar expression levels to in vivo tumor models.	[57]
Ex vivo 3D bioprinted PDX and mouse PDX models	eribulin, TAK228, doxorubicin, carboplatin, talazoparib, paclitaxel, and gemcitabine	Drug screening, common inter- and intra-cellular interactions and responses with the in vivo tumor models, spheroids Ki67 positive, active proliferation rate.	[58]
3D models of HER2-positive breast cancer	Neratinib, docetaxel	Increased innate drug resistance, expression of Akt, ERK proteins and EGFR, pEGFR, HER3 receptors, increased activity of drug metabolizing enzymes.	[59]
3D models of ER-positive/HER2-positive breast cancer	endocrine agents (tamoxifen, fulvestrant) and trastuzumab	ECM-induced pathway switches from AKT to MAPK signaling, suppression of PI3K/AKT pathway, reduced drug sensitivity, MAPK/MEK signaling.	[60]
3D MCF7 breast cancer models	Daunorubicin, Docetaxel and Arsenic Disulfide	Increased drug resistance, P-glycoprotein function.	[61]
3D models of thirteen triple-negative breast cancer cell lines	epirubicin, cisplatin, and docetaxel	Significant drug resistance.	[62]
3D collagen scaffolds with MCF7 breast cancer and glioblastoma U118-MG cells	cisplatin	Elevated levels of reactive oxygen species, reduced drug sensitivity, reduced drug uptake by the spheroids.	[63]
3D collagen-based gels reforming a ring/hollow bilayer	doxycycline and trastuzumab	Expressed similar levels of P-cadherin, vimentin, CK8, EpCAM biomarkers to human sections; destabilization of the bilayer and luminal filling of the center.	[64]
3D hyaluronic acid hydrogel scaffold/MMP peptide/nitrodibenzofuran	EGF inhibitor cetuximab	Varied EGFR expression levels of the invasive capacity of MDA-MB-231, MDA-MB-468 and MCF-7 breast cancer cell lines, differential cellular responses, different invasion capacities.	[65]
bacterial cellulose 3D scaffolds	No agent	Effective cell adhesion, proliferation, good viability and penetration rate; effectively mimics the tumor ECM.	[66]
3D scaffolds of methacrylated gelatin/adipocyte stromal cells	No agent	Human triple-negative breast cancer cell lines, HCC1806 cells and MDA-MB-231 spheroids, stiffness-dependent cellular differentiation and maturation; suppressed adipogenesis.	[67]

## Data Availability

No new data were created or analyzed in this study. Data sharing is not applicable to this article.
